# A self-consistent probabilistic formulation for inference of interactions

**DOI:** 10.1038/s41598-020-78496-8

**Published:** 2020-12-08

**Authors:** Jorge Fernandez-de-Cossio, Jorge Fernandez-de-Cossio-Diaz, Yasser Perera-Negrin

**Affiliations:** 1 Bioinformatics Department, Center for Genetic Engineering and Biotechnology (CIGB), PO Box 6162, CP10600 Havana, Cuba; 2 Systems Biology Department, Center of Molecular Immunology, PO Box 6162, CP10600 Havana, Cuba; 3 Molecular Oncology Group, Pharmaceutical Division, Center for Genetic Engineering and Biotechnology (CIGB), PO Box 6162, CP10600 Havana, Cuba

**Keywords:** Computational models, Probabilistic data networks, Epistasis, Statistics

## Abstract

Large molecular interaction networks are nowadays assembled in biomedical researches along with important technological advances. Diverse interaction measures, for which input solely consisting of the incidence of causal-factors, with the corresponding outcome of an inquired effect, are formulated without an obvious mathematical unity. Consequently, conceptual and practical ambivalences arise. We identify here a probabilistic requirement consistent with that input, and find, by the rules of probability theory, that it leads to a model multiplicative in the complement of the effect. Important practical properties are revealed along these theoretical derivations, that has not been noticed before.

## Introduction

A combination of drugs can produce synergistic effects even when administered separately in time. The first can “prepare the field” for the later action of the second, without meeting in a direct physical contact. This meaning of interaction has been practical in toxicology for the discovery of dosage combinations that better perform on clinical parameters of interest. A similar notion is in usage in epidemiology and in the construction of gene interactions networks.

Forefront high-throughput technologies are delivering interaction data relevant for the study of poorly known complex systems, like the cell. Factors and effects are often the sole kind of data released in large scale experiments by these technologies. For example, synthetic genetic arrays and gene-editing technologies, in both targeted and large-scale experiments, allows to knock out or selectively activate/repress target genes along the genome in various cell types and organisms, altering cellular process and functions^1,2^. These factors need not be in direct physical contact, while the observable effect, measured in terms of a particular biological outcome, laid far at the other end of the triggered process. The mechanisms and process structures operating in the way from factors and their effect are not observable at this experimental stage. The tacit assumption is that recurrent patterns of factor and effect provide information of the presence of cross-talk structures along the process perturbed by the factors (ambient, genes, drugs, …), in their way to the outcome of some measurable parameter (disease, blood pressure, live expectancy, cellular growth rate, fitness, transcript expression, or any other biological activity/phenotype). The above loose meaning of interaction fits to the kind of inference permitted by this kind of data, the circumstance that we focus in this manuscript.

The data in these large scale screening provide no cues of when, where and how the intermediate events interconnect^3^. Later enrichments with functional annotations and additional biological knowledge permit the systematic elucidation of higher-level principles of the cellular organization and function. The utility of theses mapping efforts has been shown across diverse prokaryotes and eukaryotes organisms including mammalian and human cells^1,4–6^. Therefore, inaccuracy at the interaction mapping stage propagates to the subsequence stages of enrichment and functional mapping^7^.

However, at the basic level, the definition of interaction remains conceptually ambivalent. The issue is not new, testimonies of everlasting debates can be traced back along more than a century^8^. Currently, diverse measures are advocated in the literature, and the concept of interaction await open for resolution.

On one side, interaction models in epidemiology gravitates around risk, i.e. the probability of a disease (effect) given the exposure factors (radiations, smoking, diet, pollution, genes, etc.). Dominant theoretical and methodological streams advocate developments from causal inference (ex. counterfactuals, potential outcomes, sufficient cause), not without controversies among scholars and philosophers^9–12^. Some epidemiologist proclaim that there is no biological rationale for calling interactions to the product terms in a regression model, arguing that they can disappear or even change sign by transforming the outcome scale (ex. logarithm)^13,14^. Others are more confident to cast sufficient-cause interactions method in complementary log regressions framework^15^. Another faction claim for broadening the scope of the casual inference school, and for a more pluralistic approach^11,12^.

On another side, ad hoc measures are adopted in the construction of large-scale genetic interaction networks. Often, regression model are written down directly in terms of the physical parameters measured in the experiment (ex. growth rate, fold change, viability, drug inhibition, lethality, etc.), with a hasty explanation for the rational of the choice^5,16–19^. Based on "genetic" grounds^20^, the double-mutant fitness is expected to be the product of the single-mutant fitness, on absence of interaction, but fitness itself exhibit a plurality of meanings, or varied in scales. A phenotype can be consistently expressed in terms of any monotonic function (logarithms, exponential, etc.) of the “original” phenotypic scale. But the product or the addition in one scale is not the same than in another. Evoking a “regression model” or a “multiplicative model” is just not enough. The concerns is not new, and the reaction should not be confined to comparisons between mathematically defined measures in term of ad hoc criteria of performance^3,7,19^. Of course, performance is the final goal, yet this path of assessment is limited to the already defined competitors, a useful but postmortem dictamen.

Striving to come out from this conceptual quandary in the direction of fundamental development, we undertake a pragmatic resolution of the concept of interaction that depart from precedent approach. In this endeavour, we do not write down in advance a mathematical definition of interaction, but advocate a concept that comply with the practical meaning and kind of input data stated above. We identify from a verbally stated definition, elementary but general probabilistic requirements for multivalued factors and dichotomous effect scenarios. Sticking to the rules of probability theory, we derive a model which is general and simple. Finally, we illustrate for a genetic interaction mapping case, how to cast the measured parameters into the language of factor and effect, so as to apply the framework just derived. Though we do not pose a causal inference approach, we detour briefly into association and causality in connection with our development.

## Motivation

**Figure 1:**
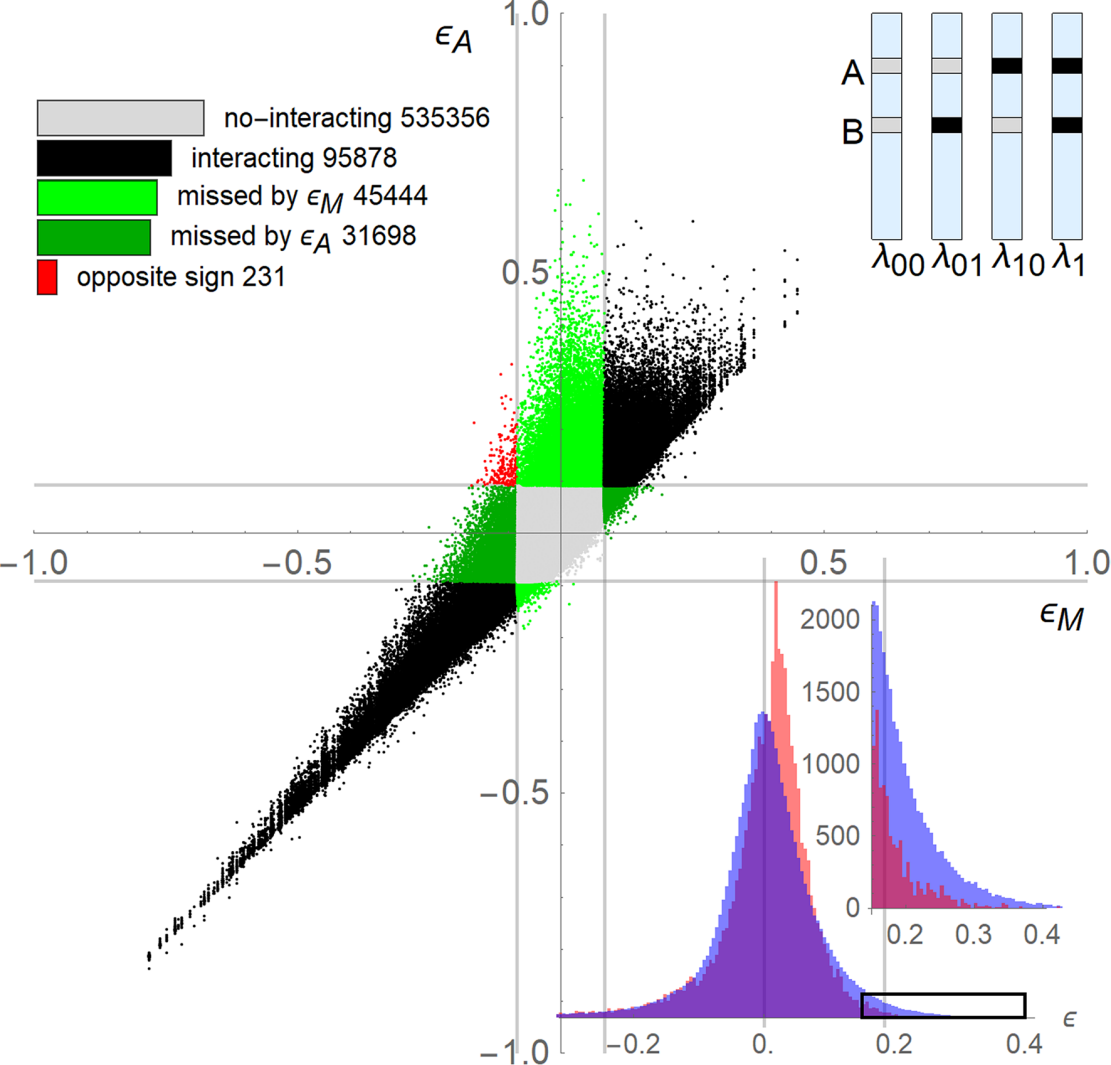
Diverging performance of two measures applied to the same interaction data. In the middle plot, genetic interaction for each pair of gene is computed with the measures $\varepsilon_{{\text{M}}} = \lambda_{11} - \lambda_{01} \lambda_{10}$ (X axis), and $\varepsilon_{{\text{A}}} = \lambda_{11} + 1 - \lambda_{01} - \lambda_{10}$ (Y axis). The color code corresponds to the bar-chart at the upper left. The parallel lines indicate the standard deviation limits, 0.083 and 0.092, for εM and εA, respectively. The count of the pairs per each category is shown in a logarithm scale in the bar-chart. The scheme at the upper right show a typical four genomes set from where interaction data are obtained for a given pair of genes. Gray and black segment of the genome denote respectively the wildtype and perturbed variant of the genes A and B. The growth rates of the corresponding yeast isogenic cultures, $\lambda_{01}$ and $\lambda_{10}$ corresponds to the single mutants, and $\lambda_{11}$ to the double mutant. The histograms at the bottom right show the distribution along the magnitude of interaction computed with measures εM (red profile) and εA (blue profile). The inset zooms the tail farther than one standard deviation toward the right tail.

Genetic interactions underlie diverse aspects of biology, including the evolution of sex, speciation, and complex disease. Simple inbred systems, such as yeast, provide an experimental format for mapping the genetic interactions networks of a cell. Genome-scale interaction studies in isogenic yeast populations, collected growth measurements from four possible mutant states for each pair of genes $A$ and $B$ (wild-type ($00$), single-mutants ($01$ and $10$), and double-mutant ($11$). A schematic representation is shown in the upper-right of Fig. 1. Genes $A$ and $B$ are regarded to interact when the growth rate $\lambda_{11}$ of the double mutant is unexpected from the growth rates $\lambda_{01}$ and $\lambda_{10}$ of the single mutants. This consent is intuitively appealing, but far from guiding to a definite physical or biological meaning, merely defers the issue to what can be regarded as a reasonable expectation of the effect in the absence of interaction. Indeed, these studies disagree on what they consider for “unexpected”, and their derived genetic interaction measures differ^7^.

Mathematical functions delivering the expectation of the combined factor effect, from the individual factor’s effect, have been named neutrality function^7^. So far, the mathematical definition of a neutrality function remain open to arbitrariness^3,7,21^. Mani et al^7^ examined properties of four reported definitions of interaction and show that the choice can dramatically alter the resulting set of genetic interactions with inconsistencies that propagates to the functional mapping. For a quick illustration of the magnitude of this impact, we compare two measures at the basic interaction networks level, of a more recent and extensive global genetic interaction studies in *Saccharomyces Cerevisiae*^1^. One of the measures, $\varepsilon_{M} = \lambda_{11} - \lambda_{01} \lambda_{10}$, use a neutrality function that multiply the single-mutants rates^1^, while the other measure, $\varepsilon_{A} = \lambda_{11} + 1 - \lambda_{01} - \lambda_{10}$, add the single-mutants rates^22^.

Figure 1 show the comparison on the Essential x Essential SGA library (See Data Availability). The initial high correlation between the most extreme negative interactions (lower-left quadrant of the plot), progressively deteriorate, due to a remarkable propensity of the measure εA to score larger positive interactions with respect to εM. Overall, the two measures have discrepancies in more than 44% of the pairs reported by one or the other measure, i.e. $\left( {\# {\text{missed}} + \# {\text{opposite}}} \right) / \left( {\# {\text{missed}} + \# {\text{opposite}} + \# {\text{interacting}}} \right)$. The superposed histograms in Fig. 1 show the distribution along the magnitude of interaction computed with both measures. A preponderance of genes with positive interaction obtained with εA are notable in the right tail, by thousands, compared to the obtained with the measure εM.

The issue is not merely on how large the deviation is, but from what and how we measure the deviation. The question of “from what?” is quantitatively answered by choosing a neutrality function. The question of “how we measure the deviation?” is another source of plural inspiration. No fundamental agreement in the answers has been achieved for neither of these questions. The commonest choices are additivity and multiplicative for the neutrality function, and arithmetic difference and the ratio for the deviation. In both cases, one choice can be converted to the other by exponentiation or logarithm. But, asking instead, what conversion is appropriate for the parameters? introduce no progress at all.

## Resolution of the concept of interaction

### Definition

Modeling controversies can become endless not because the mathematics, but because disputants might not be talking about the same thing. To keep away from such ambiguities, we commence by stating explicitly the definition of interaction we advocate.

Before jumping to write down a quantitative definition, we aim to answer the pragmatic question: what we want from what we have? The inmediate output of the large scale experiment we are focusing, are not going to explain by themselves mechanistic bases from the analysis of each factor pair, but are delivering hypotheses from loose interactions networks, that can be tested by further stages of functional analysis. Phillips^3^ quotation: “… the mutations must be interacting with one another, at least in the loose sense that they exist within pathways that both influence the same phenotype”, comply with the pragmatic meaning and the factor-effect kind of input data we aimed. Slightly re-stated for widening the context, we regard that:

Two factors interact with one another, in the loose sense that they exist within pathways that cross or interconnect, altering their individual influence to the same effect.

**Figure 2:**
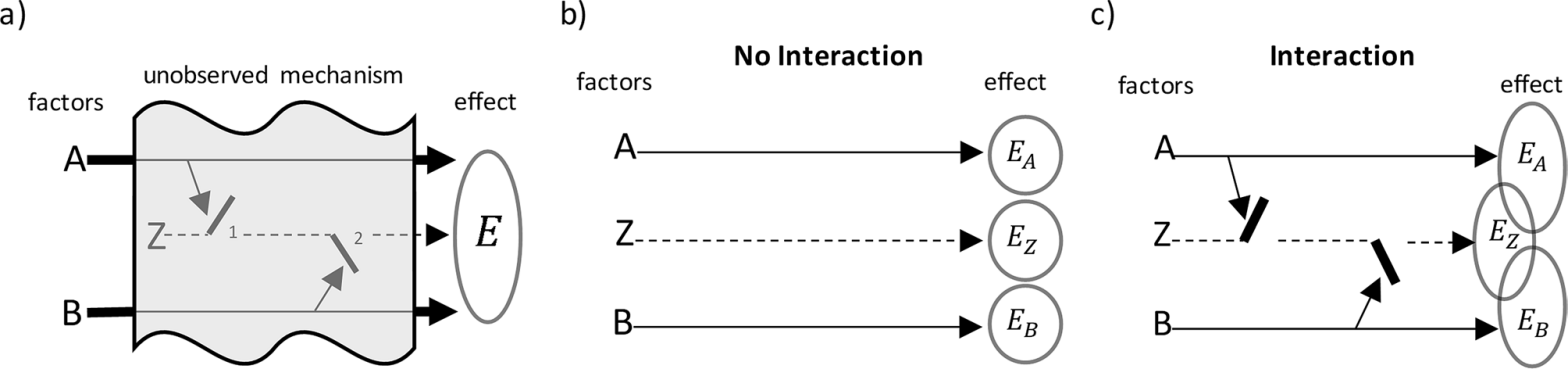
Sketch of the interaction scenarios. A and B are observed factors, Z accounts for unobserved factors and process, and E is the effect of interest. (**a**) Schematic representation of our limited information. The shaded area encloses the unobservable mechanism and background agents. Only factors A and B and the effect are observable. (**b**) No-interaction scenario. (**c**) Interaction scenario (there is a cross-over of the pathways from factors leading to the effect). (Draw with PowerPoint^23^).

Figure 2a sketch an unobserved response mechanism (shaded area) perturbed by two observed factors $A$ and $B$ leading to an effect $E$. According to the definition adopted, on absence of interaction the succession of events from each factor to the effect follow independent pathways (Fig. 2b). That is, no events triggered by factor $A$ are disturbed by events triggered by factor $B$ along the pathways of actions leading to the effect $E$. A schematic representation of interacting factors is shown in Fig. 2c. Any form of crossover or cross-talks between the pathways is regarded as interaction.

We will show how the definition, verbally stated above, become quantifiable in terms of probabilities rules.

### Mathematical formulation

We are interested in classifying the interaction or non-interaction relationships between two multivalued factors with respect to a distant dichotomous effect. An unstated and unproven assumption in previous approaches is that it is possible to infer interaction from factor and effect data alone, without considering details of the internal machinery connecting factors and effect. To prove the validity of this assumption we introduce, by the symbol $Z$, all the unobservable associated factors and process acting in the background that escape from our present scrutiny. Since $Z$ is not accessible to us from the data at reach, the effect $E$ is not fully determined by factors $A$ and $B$ under our control. A probabilistic framework is required to account for this uncertainty.

The effect due to the individual exposure to factors $A$, $B$ and $Z$ can be represented as $E_{A}$, $E_{B}$ and $E_{Z}$. In the no interaction case (Fig. 2b), the effect due to the individual or combined exposure can be represented by the logical expression $E = E_{A} \vee E_{B} \vee E_{Z}$, where $\vee$ denotes logical OR. The non-occurrence of an effect or the non-exposure to a factor, is denoted with an overbar (logical NOT). The effect $E$ is not realized only when no one of $E_{A}$, $E_{B}$ and $E_{Z}$ is realized. In other words, the negation of the effect, $\overline{E}$, is logically equivalent to $\overline{{E_{A} }} \overline{{E_{B} }} \overline{{E_{Z} }}$, where concatenation is used to denotes the logical AND. The other seven combinations (left column of Table 1), are undistinguishably from the observable $E$ . Our data is only provided in terms of $E$ or $\overline{E}$, without discerning between the possible alternative in which $E$ can be realized (left column Table 1). The logical structure $E_{A} \vee E_{B} \vee E_{Z}$ of $E$ is used as a proxy representing the unobservable mediating mechanism that involve no cross-talks. This proxy is only a temporary construct that cancels in our derivations below. The unobservables $E_{A}$, $E_{B}$, $E_{Z}$ and background $Z$ do not appear in the final equations, remaining only the observables $A$, $B$ and $E$.

**Table 1:** Logical structure $E_{A} \vee E_{B} \vee E_{Z}$ of $E$, used as a proxy representing the unobservable mediating mechanism that involve no cross-talks. The left column shows the possible combination of realization of the effect. The right column shows the single possible combination of no realization of the effect.

$E$	$\overline{E}$
$E_{A} E_{B} E_{Z}$	$\overline{{E_{A} }} \overline{{E_{B} }} \overline{{E_{Z} }}$
$$\overline{{E_{A} }} E_{B} E_{Z}$$	
$$E_{A} \overline{{E_{B} }} E_{Z}$$	
$$\overline{{E_{A} }} \overline{{E_{B} }} E_{Z}$$	
$$E_{A} E_{B} \overline{{E_{Z} }}$$	
$$\overline{{E_{A} }} E_{B} \overline{{E_{Z} }}$$	
$$E_{A} \overline{{E_{B} }} \overline{{E_{Z} }}$$	

### Rational requirement

The instances of factor $A$ are denoted by $a \in A$ and those of $B$ by $b \in B$. According to the definition, the no-interaction scenario, sketched in Fig. 2b, satisfy the following rational requirements: (i) the status of factor $B$ carries no relevant information regarding the outcome $E_{A}$, provided that the status of factor $A$ is known; (ii) the status of factor $A$ and the occurrence of effect $E_{A}$ carry no relevant information regarding $E_{B}$, provided that the status of factor $B$ is known; and similarly, (iii) the status of factors $A$ and $B$, and effects $E_{A}$ and $E_{B}$ joined, carry no relevant information regarding $E_{Z}$ Each of the assertions i, ii, and iii has a definite translation in the language of probability theory, that corresponds to the following equalities:

1 $$\begin{aligned} & {\text{(i}})\quad \Pr \left( {E_{A} {|}a b} \right) = \Pr \left( {E_{A} {|}a} \right) \\ & {\text{(ii}})\quad \Pr \left( {E_{B} {|}E_{A} a b} \right) = \Pr \left( {E_{B} {|}b} \right) \\ & {\text{(iii}})\quad \Pr \left( {E_{Z} {|}E_{A} E_{B} a b} \right) = \Pr \left( {E_{Z} } \right) \\ \end{aligned}$$

where (1) stands for every $a \in A$ and $b \in B$, and for every combination replacing instances of $E_{{{\text{factor}}}}$ at the right of the vertical bar by the complement $\overline{E}_{{{\text{factor}}}}$. For example, in $\Pr \left( {E_{B} {|}\overline{E}_{A} a b} \right) = \Pr \left( {E_{B} {|}b} \right)$ in ii.

Therefore, the equalities in (1) are quantitative translations of the rational requirements i, ii, and iii, which in turn comply with the verbal definition of interaction. Everything that follows are derived from (1) and the rules of probability theory.

### A necessary condition

The product rule of probability theory, i.e. $\Pr \left( {x y z} \right) = \Pr \left( x \right)\Pr \left( {y{|}x} \right)\Pr \left( {z{|}x y} \right)$, and the requirements (1) imply the following factorization:

2 $$\Pr \left( {E_{A} E_{B} E_{Z} |a b} \right) = \Pr \left( {E_{A} |a} \right)\Pr \left( {E_{B} {|}b} \right)\Pr \left( {E_{Z} } \right)$$

for all $a \in A$ and ${ }b \in B$. Like with (1), equality (2) is also valid for any of the eight combinations in Table 1. However, since the data only account for the realization of the observed effect, i.e. $E$ or $\overline{E}$, the seven combinations accounting for the observed $E$ are not individually discernable from the observations, while only $\overline{{E_{A} }} \overline{{E_{B} }} \overline{{E_{Z} }}$ can be, since it is equal to $\overline{E}$. Hence

3 $$\Pr \left( {\overline{E}{|}a b} \right) = \Pr \left( {\overline{{E_{A} }} {|}a} \right)\Pr \left( {\overline{{E_{B} }} {|}b} \right)\Pr \left( {\overline{E}_{Z} } \right),{ }\forall a \in A,{ }b \in B$$

is the sole necessary condition for non-interacting factors $A$ and $B$, accessible to us. Here $\Pr \left( {\overline{E}{|} \cdots } \right) = 1 - \Pr \left( {E{|} \cdots } \right)$, is the probability of the complement of the effect.

The factorization in the right side of (3) is the expected frequency of no-effect in the absent of interaction. Requirement (3) restrict the space of probability distributions allowed for the probability of the effect given non-interacting factors. Since this is crucial for our subsequent derivations and purposes, we denote this particular factorization by ${\mathcal{N}}\left( {\overline{E}{|}a b} \right)$, and reserve to it the name neutral model. Hence a necessary condition for no interactions (3) can be stated by $\Pr \left( {E{|}a b} \right) = {\mathcal{N}}\left( {E{|}a b} \right)$, with the understanding that ${\mathcal{N}}\left( {E{|}a b} \right) = 1 - {\mathcal{N}}\left( {\overline{E}{|}a b} \right)$.

This path of reasoning departs from previous approaches appealing to neutrality functions or casual inference arguments. The factorization (3) is a consequence of the rules of probability theory, given that (1) are satisfied. Equalities (1), in turn, followed from the verbal definition of interaction. Still, the form of the neutral model presented in (3) does not allows practical evaluation, since it is not expressed in terms of observables, so far.

### Interaction measure

We arrived to the neutral model (3) through a path not related to log-linear forms. Now we will look for the connections of (3) with logarithm forms. The logarithm of the probability of the complement of the effect, at interaction or no-interaction scenarios, can be expressed in the form

4 $$\log \left\{ {\Pr \left( {\overline{E}{|}x y} \right)} \right\} = \mu + \alpha_{x} + \beta_{y} - \delta_{xy} ,{ }\forall x \in A,{ }y \in B$$

The identity can be conveniently shown by substituting (5), (6) and (7) in (4).

5 $$\mu = \log \Pr \left( {\overline{E}{|}\overline{a}{ }\overline{b}} \right)$$

6 $$\alpha_{x} = \log \frac{{\Pr \left( {\overline{E}{|}x{ }\overline{b}} \right)}}{{\Pr \left( {\overline{E}{|}\overline{a}{ }\overline{b}} \right)}}, \beta_{y} = \log \frac{{\Pr \left( {\overline{E}{|}\overline{a}{ }y} \right)}}{{\Pr \left( {\overline{E}{|}\overline{a} \overline{b}} \right)}}$$

7 $$\delta_{xy} = \alpha_{x} + \beta_{y} - \log \frac{{\Pr \left( {\overline{E}{|}x y} \right)}}{{\Pr \left( {\overline{E}{|}\overline{a}{ }\overline{b}} \right)}}$$

In the particular non-interaction case, the requirement (3) implies that

8 $$\begin{aligned} \mu & = \log \Pr \left( {\overline{E}_{A} {|}\overline{a}} \right) + \log \Pr \left( {\overline{E}_{B} {|}\overline{b}} \right) + \log \Pr \left( {\overline{E}_{Z} } \right) \\ \alpha_{x} & = \log \Pr \left( {\overline{E}_{A} {|}x} \right) - \log \Pr \left( {\overline{E}_{A} {|}\overline{a}} \right) \\ \beta_{y} & = \log \Pr \left( {\overline{E}_{B} {|}y} \right) - \log \Pr \left( {\overline{E}_{B} {|}\overline{b}} \right) \\ \delta_{xy} & = 0 \\ \end{aligned}$$

Hence, absence of interaction implies a log-linear form of the probability distribution in the *complement* of the effect, and $\delta_{ab} \ne 0$ is required for detectable interactions.

### Interaction hypothesis

Fixing $\delta_{xy} = 0$ in (4) implies the log-linearity of ${\mathcal{N}}\left( {\overline{E}{|}x y} \right)$, that is:

9 $${\mathcal{N}}\left( {E{|}x y} \right) = 1 - \exp \left( {\mu + \alpha_{x} + \beta_{y} } \right),{ }\forall x \in A,{ }y \in B$$

If $\delta_{ab} > 0$ there is positive interaction, since the probability of the effect of the combined factors, $\Pr \left( {E{|}a{ }b} \right)$, is greater than the expected,${\mathcal{N}}\left( {E{|}a b} \right)$, as can be corroborated from (4) and (9). If $\delta_{ab} < 0$ there is negative interaction, since $\Pr \left( {E{|}a{ }b} \right) < N\left( {E{|}a b} \right)$. Hence, any monotonous function of $\delta_{ab}$ can be used as a measure of interaction. We can compare the null hypothesis $\delta_{ab} = 0$ versus the interaction hypothesis $H_{1}$:

10 $$H_{0} :\delta_{ab} = 0, H_{1} :\delta_{ab} \ne 0$$

Notice that requirement (3) entails practical limitations since it is expressed in terms of non-observables $E_{A}$, $E_{B}$ and $E_{Z}$. The measure of interaction $\delta_{ab}$, however, can be fully expressed in terms of observables from (6) and (7) by

11 $$\delta_{ab} = \log \frac{{\Pr \left( {\overline{E}{|}a \overline{b}} \right)\Pr \left( {\overline{E}{|}\overline{a} b} \right)}}{{\Pr \left( {\overline{E}{|}\overline{a} \overline{b}} \right)\Pr \left( {\overline{E}{|}a b} \right)}}$$

where $a, \overline{a} \in A$ and $b, \overline{b} \in B$. The null hypothesis $\delta_{ab} = 0$ turned to be the model multiplicative in the complement of the effect, which is equivalent to the Finney’s independent action model $q_{11} q_{00} = q_{01} q_{10}$, where $q_{xy} = \Pr \left( {\overline{E}{|}x y} \right)$, and $x,y \in \left\{ {0,1} \right\}$^24,25^. Lee^15^ provided instructions to perform such a regression using existing statistical software.

### Neutral model

Faithful to the rules of probability theory, we derive now an equivalent expression of (3) in terms of observables only. Multiplying both sides of (3) by $\Pr \left( {b{|}a} \right)$, summing over $b \in B$, and doing the same but with $\Pr \left( {a{|}b} \right)$ over $a \in A$, yields

12 $$\begin{gathered} \Pr \left( {\overline{E}{|}a} \right) = \Pr \left( {\overline{E}_{A} {|}a} \right)\Pr \left( {\overline{E}_{B} {|}a} \right)\Pr \left( {\overline{E}_{Z} } \right) \hfill \\ \Pr \left( {\overline{E}{|}b} \right) = \Pr \left( {\overline{E}_{A} {|}b} \right)\Pr \left( {\overline{E}_{B} {|}b} \right)\Pr \left( {\overline{E}_{Z} } \right) \hfill \\ \end{gathered}$$

where $\Pr \left( {\overline{E}_{B} {|}a} \right) = \mathop \sum \limits_{b \in B} \Pr \left( {\overline{E}_{B} {|}b} \right)\Pr \left( {b{|}a} \right)$ and $\Pr \left( {\overline{E}_{A} {|}b} \right) = \mathop \sum \limits_{a \in A} \Pr \left( {\overline{E}_{A} {|}a} \right)\Pr \left( {a{|}b} \right)$, as warranted by the requirements (1) (see also Supplementary Eq. 1). From (12) the factorization (3) can be written

13 $${\mathcal{N}}\left( {\overline{E}{|}a b} \right) = \frac{{\Pr \left( {\overline{E}{|}a} \right)\Pr \left( {\overline{E}{|}b} \right)}}{{\Pr \left( {\overline{E}_{A} {|}b} \right)\Pr \left( {\overline{E}_{B} {|}a} \right)\Pr \left( {\overline{E}_{Z} } \right)}}$$

Expanding the product of summations in the denominator and using (3) yields

14 $$\begin{aligned} & \Pr \left( {\overline{E}_{A} {|}b} \right)\Pr \left( {\overline{E}_{B} {|}a} \right)\Pr \left( {\overline{E}_{Z} } \right){ } \\ & \quad = \Pr \left( {\overline{E}_{Z} } \right)\left\{ {\mathop \sum \limits_{x \in A} \Pr \left( {\overline{{E_{A} }} {|}x} \right)\Pr \left( {x{|}b} \right)} \right\}\left\{ {\mathop \sum \limits_{y \in B} \Pr \left( {\overline{E}_{B} {|}y} \right)\Pr \left( {y{|}a} \right)} \right\} \\ & \quad = \mathop \sum \limits_{x \in A, y \in B} \Pr \left( {\overline{{E_{A} }} {|}x} \right)\Pr \left( {\overline{E}_{B} {|}y} \right)\Pr \left( {\overline{E}_{Z} } \right)\Pr \left( {x{|}b} \right)\Pr \left( {y{|}a} \right) \\ & \quad = \mathop \sum \limits_{x \in A,y \in B} \Pr \left( {\overline{E}{|}x y} \right)\Pr \left( {x{|}b} \right)\Pr \left( {y{|}a} \right) \\ \end{aligned}$$

Substituting (14) in the denominator of (13) demonstrate that the neutral model ${\mathcal{N}}\left( {\overline{E}{|}a b} \right)$ satisfy:

15 $${\mathcal{N}}\left( {\overline{E}{|}a b} \right) = \frac{{\Pr \left( {\overline{E}{|}a} \right)\Pr \left( {\overline{E}{|}b} \right)}}{{\mathop \sum \nolimits_{x \in A,y \in B} \Pr \left( {\overline{E}{|}x y} \right)\Pr \left( {x{|}b} \right)\Pr \left( {y{|}a} \right)}}, a \in A,b \in B$$

As noted from (3) and its derived (11), (15), the neutral model, does not relate physical magnitudes directly, but only through the probabilities of the effect. The form of the model is valid independently of how the factors and effect can be defined in the application domain. Hence, the neutral model “knows” how to measure interactions before casting the physical magnitudes into factors and effects concepts. On the contrary, a neutrality function predicts the expected effect directly in terms of physical magnitudes, for example fitness, growth rate, etc. Hence, the form of the neutrality function depends on the application domain, and as so, it has no universal or unifying validity, and are chosen by ad hock intuitive criteria. We will return later to the neutral model and the application-specific castings.

## Theoretical implications

### Log linearity of the neutral model

We already shown, Eqs. (4)–(8), that absence of interaction implies a log-linear form of the probability distribution in the complement of the effect. We will demonstrate now the converse, that a log-linear function in the complement of the effect is a neutral model. It is not obvious, from the weird looking expression at the right side, that a log-linear form satisfies the equality (15). We then assert that $\Pr \left( {\overline{E}{|}x y} \right) = \exp \left( {\mu + \alpha_{x} + \beta_{y} } \right)$, and after proper substitutions and arrangements in the right side, it becomes equal to $\Pr \left( {\overline{E}{|}x y} \right)$, the left side.

The numerator in the right side of (15) becomes

$$\begin{gathered} \Pr \left( {\overline{E}{|}a} \right) = \mathop \sum \limits_{y \in B} \Pr \left( {\overline{E}{|}a y} \right)\Pr \left( {y{|}a} \right) = \exp \left( {\mu + \alpha_{a} } \right)\mathop \sum \limits_{y \in B} \exp \left( {\beta_{y} } \right)\Pr \left( {y{|}a} \right) \hfill \\ \Pr \left( {\overline{E}{|}b} \right) = \mathop \sum \limits_{x \in A} \Pr \left( {\overline{E}{|}x b} \right)\Pr \left( {x{|}b} \right) = \exp \left( {\mu + \beta_{b} } \right)\mathop \sum \limits_{x \in A} \exp \left( {\alpha_{x} } \right)\Pr \left( {x{|}b} \right) \hfill \\ \end{gathered}$$

The denominator in (15) becomes

$$\mathop \sum \limits_{x \in A,y \in B} \exp \left( {\mu + \alpha_{x} + \beta_{y} } \right)\Pr \left( {x{|}b} \right)\Pr \left( {y{|}a} \right) = \exp \left( \mu \right)\mathop \sum \limits_{x \in A} \exp \left( {\alpha_{x} } \right)\Pr \left( {x{|}b} \right)\mathop \sum \limits_{y \in B} \exp \left( {\beta_{y} } \right)\Pr \left( {y{|}a} \right)$$

The summation terms cancel in the numerator and denominator of (15), yielding $\exp \left( {\mu + \alpha_{a} + \beta_{b} } \right)$, that is, $\Pr \left( {\overline{E}{|}a b} \right) = {\mathcal{N}}\left( {\overline{E}{|}a b} \right)$.

Therefore, a log-linear function in terms of $x \in A$, and $y \in B$ is a neutral model in the complement of the effect.

### Relation to other conventional models

We show first how the conventional additive and multiplicative models in epidemiology can be related to the model multiplicative in the complement of the effect. Later we will see how to derive the additive model as an approximation.

Equating to (1) the exponential of $\delta_{ab}$ in (11) , multiplying by the denominator, and expanding the products of $1 - \Pr \left( {E{|} \cdot } \right)$ yields:

16 $$\Pr \left( {E{|}a b} \right) + \Pr \left( {E{|}\overline{a} \overline{b}} \right) - \Pr \left( {E{|}a b} \right)\Pr \left( {E{|}\overline{a} \overline{b}} \right) = \Pr \left( {E{|}\overline{a} b} \right) + \Pr \left( {E{|}a \overline{b}} \right) - \Pr \left( {E{|}\overline{a} b} \right)\Pr \left( {E{|}a \overline{b}} \right)$$

Rearranging equality (16) yields:

17 $$\left( {R_{ab} + R_{{\overline{a}\overline{b}}} } \right) - \left( {R_{{\overline{a}b}} + R_{{a\overline{b}}} } \right) = R_{ab} R_{{\overline{a}\overline{b}}} - R_{{\overline{a}b}} R_{{a\overline{b}}}$$

where $R_{ij} = \Pr \left( {E{|}i j} \right)$ is the usual notation in epidemiology for risk, the probability of disease ($E$), by exposure to factors $i \in \left\{ {a,\overline{a}} \right\}$ and $j \in \left\{ {b,\overline{b}} \right\}$. Hence, the requirement $\delta_{ab} = 0$ of no interaction is satisfied when the above equality holds. The conventional additive law accounts for equating the left side to cero, and the conventional multiplicative law accounts for equating the right side to cero. When both laws are equal to zero, equality (17) holds, and the three models -additive, multiplicative, and multiplicative in the complement of the effects- agree to discard interaction (true negative). When only one of the sides is equal to cero, the corresponding law discard a true interaction (false negative). When both sides are different from cero but equal, both laws are delivering false interactions (false positive). When both sides are different from cero and equality (17) does not holds, the three laws are delivering true interactions (true positive). This show that the multiplicative rule is the bias-size for correction of the additive rule, and vice versa.

Assuming that $\Pr \left( {E{|} \cdot } \right)$ are small in (16), the cross products can be neglected. Dividing both sides by $\Pr \left( {E{|}\overline{a} \overline{b}} \right)$ yields the additive model^26^:

18 $$\frac{{R_{ab} }}{{R_{{\overline{a}\overline{b}}} }} = \frac{{R_{{\overline{a}b}} }}{{R_{{\overline{a}\overline{b}}} }} + \frac{{R_{{a\overline{b}}} }}{{R_{{\overline{a}\overline{b}}} }} - 1$$

Thus, the model multiplicative in the complement of the effect justifies the additive model as an approximation valid for low risk regimes, as has been previously settled^27^.

Multiplicative or additive models, advocated for long in the epidemiology literature, can be useful approximations in some domains, which might explain why they remain pervasive in the interaction field.

### Cross-product terms and interaction

We demonstrated that the cross-product term $\delta_{xy}$ of the log-linear form of the probability on the *complement* of the effect convey an interaction meaning. Can we say the same for the probability on the effect? Nothing in mathematic forbid us to write the logarithm of $\Pr \left( {E{|}x y} \right)$ in the form

19 $$\log \left\{ {\Pr \left( {E{|}x y} \right)} \right\} = \mu^{\prime} + \alpha^{\prime}_{x} + \beta^{\prime}_{y} - \delta^{\prime}_{xy} ,{ }\forall x \in A,{ }y \in B,$$

and follows the analog of Eqs. (4)–(7), with $E$ instead of $\overline{E}$. We can even calculate the cross-product term $\delta ^{\prime}_{xy}$ by the analog of Eq. (11), evaluated in $E$. However, there is nothing analog to (8) supporting that $\delta ^{\prime}_{xy} = 0$ is a necessary condition derived from the definition of interaction advocated in this manuscript. The result in (8) follows from the factorization (3), which is valid only on the *complement* of the effect, $\overline{E}$, but not on $E$, as was previously shown from (1) and Table 1. The opposite necessarily constitutes a departure from the rules of probability theory.

A real-life scenario of an evident interaction case, where $\delta_{xy} \ne 0$ and $\delta ^{\prime}_{xy} \ne 0$, is approached in Supplementary Discussion. A simulation demonstrating that the “interaction” terms $\delta_{xy}$ and $\delta ^{\prime}_{xy}$ differ in general are shown in Supplementary Fig. S1.

Let now see how disappointing is the strong condition $\delta_{xy} = \delta ^{\prime}_{xy} = 0$. Equality (17) is satisfied since $\delta_{xy} = 0$. The right side of (17) become cero, since $\delta ^{\prime}_{xy} = 0$. The only way that the left side equal cero is with $\alpha^{\prime}_{x} = 0$ or $\beta^{\prime}_{y} = 0$. But both imply that the probability of the effect depends on a single factor, a trivial case of no-interaction, that can be anticipated even without caring on conceptual issues.

Therefore, the cross-product term $\delta ^{\prime}_{xy}$ in the logarithm of the probability of the effect, (19), is not a consistent measure of interaction. According to this, the model of interaction multiplicative in the effect necessarily departs from the definition of interaction and the rules of probabilities theory, and is not generally valid.

Gene-interaction studies wrote down different “interaction” functions in terms of fitness^1,7,22^. Indeed, many of such functions can be defined. But as shown by Eqs. (4)–(7), every real value function ($\ne 0$) for binary-valued x, y can be expressed in the log-form $\mu + \alpha {\text{x}} + \beta {\text{y}} + \delta {\text{xy}}$. Those functions for which the cross-product term equal cero ($\delta = 0$) are log-linear, but the fitness-values space nullifying $\delta$ depends on the function chosen. Shall we call “interaction” to the cross-product term $\delta$ of any such arbitrarily chosen function? Certainly not, otherwise we cannot avoid inconsistent and contradicting calls for interaction.

In general, just because the log of a mathematical function $f\left( {x,y} \right)$ can be represented in the form $\mu + \alpha {\text{x}} + \beta {\text{y}} + \delta {\text{xy}}$, does not entail us to blindly attach a real-world interaction meaning to the term $\delta$. It depends on what function we are talking about, and the reality we are modelling. Unfortunately, this subtle confusion pervades the subject for long.

Therefore, a criterium connected to reality precede the question of whether the cross-product term represent interaction; otherwise, the call for interactions remain ill posed. We got a criterium leading to (3), plainly raised from our definition of interaction, by asking and understanding on what model we are entailed to represent that reality. On doing so, we demonstrated that the probability on the *complement* of the effect, ${\text{Pr}}(\overline{E}| x y)$, can instantiate the function $f\left( {x,y} \right)$, and then, that the cross-product term of the log-form of *this* function can be interpreted as an interaction term.

### Mechanistic details

The notation $E_{A}$, $E_{B}$, $E_{Z}$ and $Z$ summarize the unobserved mechanistic structure in the logical framework allowed by our observations. All these structures cancel in the derivation (3)–(15) of the neutral model. Therefore, departure from $\delta_{ab} = 0$ ensures that there is no possible separation of the pathways leading to effect $E$ that can be independently associated to the factors $A$ and $B$, and requirement (3) cannot hold for any resolution of the effect $E$ into a disjunction $E_{A} \vee E_{B} \vee E_{Z}$. Fortunately, because of this, searching for a particular splitting $E_{A}$, $E_{B}$ and $E_{Z}$ of the effect $E$ satisfying (3), that might not even exist, is not required for the diagnosis of interaction, provided $\delta_{ab} \ne 0$. In other words, knowledge of the “mechanistic” structure of the events between the factors and the effects are not required to test for interaction. This was so far a tacit assumption, now demonstrated as valid.

This is of crucial importance in practice. For example, in the large scale interaction studies performed to build interconnected maps of simpler organisms^1^, millions of gene pairs are tested but only a small fraction interacts. This already daunting task would be impossible if the molecular mechanisms mediating each possible pair were required a priori to select the “appropriate” null model to detect interaction. Instead, the presence of interactions can be diagnosed by (10). Subsequent experiments to discern interaction mechanisms (when, where and how) can be specifically targeted toward the promising interacting pairs, without wasting efforts in the rejected pairs.

## Genetics interactions into the framework

### Casting genetic interactions

We derived a neutral model in terms of general abstract concepts of factors and effect, without explicit reference to physical parameters. In this sense, it is a unifying theoretical framework. But, to make practical application of this framework, it is required to land the model into actual data scenarios. The model obtained here from basic principles, though accepted in toxicology, is ignored or undervalued by the dominant genetic networks literature^1,7,14,28^. This can be in part because to cast the physical parameters (growth rate) into the probability framework of factor and effect concepts is not straightforward. The example in the domain of genetic interaction introduced in Motivation serve the purpose to illustrate how this translation can be performed. After casting the model into this domain, we demonstrate that one of the measures we were comparing, additive on fitness εA, can be derived from the model multiplicative in the complement of the effect.

Several genome-scale interaction studies have been conducted in yeast (*Saccharomyces Cerevisiae*). Even when addressing the same interaction question, and advocate the same null multiplicative model $f_{11} f_{00} = f_{01} f_{10}$ on fitness $f$, their definitions of fitness differ, and so are their predictions^7^. The sub-indices correspond to wild-type ($00$), single-mutants ($01$ and $10$), and double-mutant ($11$). For example:

Jasnos et al.^22^ assayed growth curves of the resulting progeny of 639 randomly crossed pairs of isogenic individuals with deletions performing slow growth rates in one of 758 genes. These authors defined fitness $f$ by the factor $e^{\lambda }$, of a population growing continuously at a rate $\lambda$, and chose the null model as the log-fitness scale $\varepsilon = \left( {\lambda_{00} + \lambda_{11} } \right) - \left( {\lambda_{01} + \lambda_{10} } \right)$, which become additive on rates.

Onge et al.^20^ studied the interaction of 650 double-deletion strains, corresponding to pairings of 26 non-essential genes that confer resistance to the DNA-damaging agent methanesulfonate (MMS). These authors defined fitness of each deletion strain directly by its duplication rate $\lambda$, relative to that of wild type, i.e. $f = \lambda$. The null model takes the form $\varepsilon = \lambda_{11} - \lambda_{01} \lambda_{10}$, where $\lambda_{00} = 1$.

Costanzo et al.^1^ wired the most extensive global genetic interaction network in *Saccharomyces Cerevisiae*, with over 23 million double mutants involving 5 416 different genes, including the first large-scale interaction network comprising ~ 120 000 pairs of essential genes. This hallmark works revealed the first comprehensive global functional genetic landscape. These authors defined fitness proportional to colony growth rate relative to that of wild type, i.e. $f \propto \lambda$. Like Onge et. al., the null model takes the form $\varepsilon = \lambda_{11} - \lambda_{01} \lambda_{10}$.

Can the measures used in these studies be casted in terms of the model derived here? To address this interrogation in a unified manner, the experimental problem originally contextualized in terms of genes and fitness, need to be reformulated here in terms of factors and effect.

Let $\lambda$ denotes the average rate of cell duplication per unit of time. The probability for a strain $xy$ that cell duplicates at least once in a lapse of time $t$ (i.e. $E \equiv n > 0$) can be modeled according to^29^ by

20 $$\Pr \left( {E{|}xy} \right) = 1 - e^{{ - \lambda_{xy} t}}$$

In this case $x$ and $y$ might denote gene variants at two loci $A$ and $B$, respectively. We choose, without losing generality, the duplication average time of wild-type strain as the unit of time, i.e. $\lambda_{00} = 1$. The measure for genetic interaction by substituting (20) in (11) yields

21 $$\delta_{ab} = \left( {1 + \lambda_{ab} } \right) - \left( {\lambda_{{\overline{a}b}} + \lambda_{{a\overline{b}}} } \right)$$

By posing the genetic interaction problem in terms of probabilities of factors and effect, the equality (21) show that the multiplicative model in the complement of the effect imply additivity of duplication rates. This is the measure used by Jasnos et al.^22^ denoted εA in Motivation, now supported from basic principles. As matter of fact, these authors provided a brief but appealing justification of their choice.

### Gene-hubs hunting

We take a glance on the functional mapping implications, to settle some ground on the application arena. It is not our purpose to dwell deeper on functional mapping, having others devised and championed. Further, diverse methods have been developed for discovering and visualization of functional and organizational features of the cell. Though ingenious and successful in their purpose, they are ad hoc devices in a large part, that preclude their use as standard-gold for performance assessment of others methods. We have no answer to … which one to choose for benchmark? In Motivation we illustrated the diverging performance of εM and εA in mapping interaction networks, just by counting interactions and no-interactions, which is a factual fair comparison, without the introduction of third-party intricated post-processing bias. Similarly, here we use the counting factual method to assess some basic elements of functional implication. We compare the number of interactors per genes, and look for biological meanings for the genes exhibiting distinct connection patterns regarding the measures.

Cellular process in an organism can accounts for the integrated and concerted activity of specific “gene constellations, forming a complex hierarchical web of molecular interactions. It is a well-known fact that most genes interact with a limited number of other genes, whereas only a smaller set of genes interacts with many other genes (network hubs)^30^. Perturbations of genes-hubs are expected to have a major fitness impact, i.e. more essential^31–33^. These genes play prominent roles in the characteristics and development of diseases^34^.We will explore how the gene degrees (# of interactors) are distributed by the two measures, and the correspondence with biological evidence.

**Figure 3:**
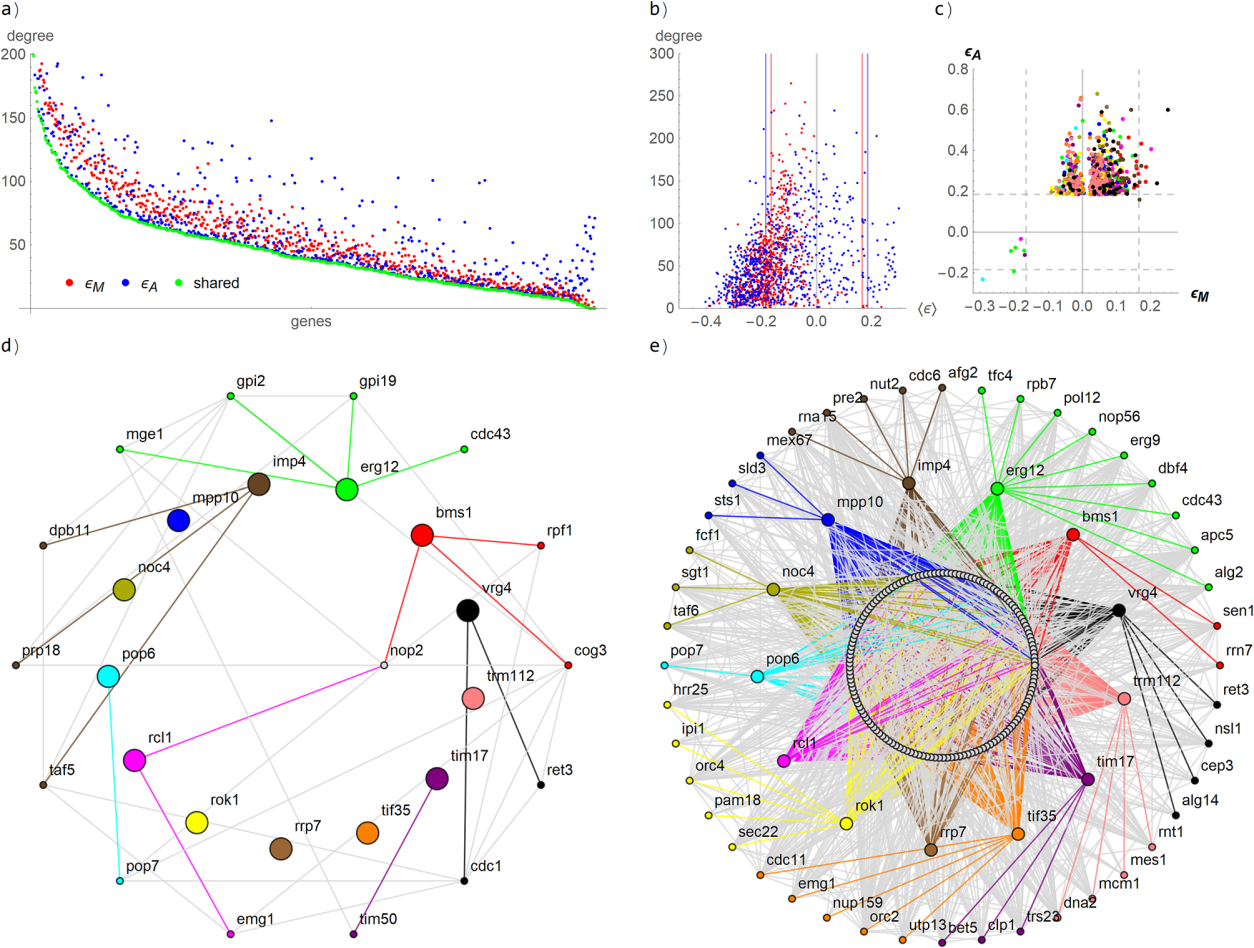
Distribution of # of interactors per genes in the Essential × Essential library. (**a**) At each gene, three dots (red, blue and green) are located according to the number of interactors obtained by εM, εA and by both, respectively. (**b**) Number of interactors (Y axis) vs. the average interaction score per genes (X axis). Red dots are computed with εM and blue dots with εA. The red and blue vertical lines are the two standard deviation limits, respectively. (**c**) Comparison of the interactions scores εM and εA for candidate hubs of Table 2. (**d**) and (**e**) Interaction network of the candidates’ hubs of Table 2, including the connections between the interactors. The hubs are located in the middle ring with larger dots. The interactors that are not connected to more than one hub are in the outer ring. The rest of interactors are in the inner ring. The hub-connections has the same color of the corresponding hubs. The other connections are in light gray. (**d**) Interaction network as computed by εM. (**e**) Interaction network as computed by εA. The dot colors are consistently used in (**c**–**e**).

In Fig. 1 above, the measures agree on about 60% of the detected interactions in the Essential x Essential library^1^. However, a preponderance of genes with larger positive interaction is apparent. The histograms in Fig. 1 show that the preponderance in the order of thousands for εA > 1.5 s.d. Now, we explore how the two measures distribute gene degrees (# of interactors) per average interactions score of the corresponding gene interactors (Fig. 3b). There is no large divergence between both measures for the negative interactions. However, toward the positive scores, the εA measure (blue dots) predominate with larger degrees, in particular for values greater than one and two standard deviations (right of the vertical blue line).

Genes with ten or more interactors, obtained at least by any of the measures εM or εA, are plotted in Fig. 3a. The genes are ordered in the X-axis by the number of interactors shared by both measures. At each gene position, three dots (red, blue and green) are located by the Y-axis according to the number of interactors obtained by εM, εA, and the number of common interactors they predict (shared), respectively. As can be appreciated from this plot, the genes not only show similar number of interactors (red and blue dots), but they share the identity of most of the interactors (green), otherwise the green dots have had appeared separated down from the red and blue dots. However, again, a preponderance of blue dots above the red dots is pretty apparent in Fig. 3a, indicating that the measure εA report more interactors per genes than εM.

With these preliminary evidences, we find relevant for the comparison of gene degrees, to be more restricting in regarding interactions when the magnitude of the measure is larger than two standard deviations of the values obtained for the complete library (i.e. ε > 2 s.d.). This restriction is more conservative in assuming approximately that less than 5% of the pairs interact (with one standard deviation the number rise to about 33%). Then we apply a deliberate but simple criterium to select candidate gene-hubs obtained from one measure and missed by the other. It is a fair symmetric criterium, based on simple counting. We look for genes that according to one measure has less than 10% of the number of interactions captured by the other measure. The candidate hubs so captured from the Essential x Essential library are listed in Table 2.

**Table 2:** List of genes that according to one measure has less than 10% of the number of interactions only captured by the other measure. The interactions scores were computed from the Essential x Essential library with the measures $\varepsilon_{M} = \lambda_{11} - \lambda_{01} \lambda_{10}$ and $\varepsilon_{A} = \lambda_{11} + 1 - \lambda_{01} - \lambda_{10}$.

Candidate hubs	# of interactors ($\varepsilon_{M}$)	# of interactors ($\varepsilon_{A}$)	# of common interactors
mpp10	0	72	0
trm112	0	71	0
erg12	4	65	1
tif35	0	64	0
noc4	0	57	0
rok1	0	55	0
bms1	3	50	3
vrg4	2	47	2
rcl1	2	45	1
tim17	1	43	0
imp4	3	38	2
rrp7	0	37	0
pop6	1	23	1

The measure εM does not deliver genes satisfying this tenfold criterium, neither even a twofold one. Supplementary Table S1 list the set of genes with a weaker 1.5-fold criterium. Even in this set of genes scantily favoring εM, the measure εA predicted more than 57% of the interactors predicted by εM (last two columns of Table S1). The magnitudes of the interactions of the genes in Table S1, obtained by both measures, are contrasted in Supplementary Figure S2a. A linear correspondence is pretty apparent. The network of these eight hubs are created according to both measures in Supplementary Figure S2b-c.

Notably in contrast, 13 genes satisfied the tenfold criterium in favor of εA. Further, the measure εM predicted for these genes less than 3% of the interactors predicted by εA. The magnitudes of the interactions of the candidate hubs of Table 2, obtained by both measures, are contrasted in Fig. 3c. The great majority of εA-interactions are positive, which seem to cover a “blind zone” of εM-measure.

### Candidate hubs biology

The interaction networks in-between candidate hubs and interactors, obtained by εM and εA, are show in Fig. 3d–e. The interaction network obtained by εM (Fig. 3d) is pretty sparse. Six of the 13 candidate hubs have no interactors, and the remaining seven display from one to four interactors. In clear contrast, the interaction network obtained by εA is strikingly much denser (Fig. 3e). This contrast is not corresponded the other way around, even by the weaker 1.5 criterium (genes which according to εM have more than 1.5 times interactors than εA) (Figure S2b-c), despite favoring εM (tenfold for εA vs. 1.5-fold for εM).

The 13 candidate hubs, exclusively surfaced by εA, have a significant number of experimentally interactions verified in the Saccharomyces Genome Database (SGD) (http://www.yeastgenome.org/). For instance, SGD listed more than 100 physical and genetic interactions (GI) for genes mpp10 (GI = 23), tif35 (GI = 44), noc4 (GI = 28), and rrp7 (GI = 41), (Supplementary Table S2), whereas εM found no genetic interactions with these genes. A ribosome biogenesis factor, bms1, is another salient hub annotated in SGD with more that 400 interactors (GI = 360). This hub is poorly corresponded by εM with barely 3 interactors, while εA detected 50, including the 3 ones of εM. Furthermore, the Temperature Sensitive (TS) alleles of these 13 hubs significantly decrease yeast growth fitness (i.e. between 0.2037 and 0.4778) as expected for a highly interconnected hub protein^31,32^.

The 13 candidate hubs comprise diverse molecular functions and cellular components (Supplementary Table S2). For instance, tif35 and tim17 are essential components of molecular complexes partaking protein translation and mitochondrial import channel structure^35,36^. Erg12 is an essential gene coding for a Mevalonate kinase which is involved in the biosynthesis of isoprenoids and sterols^37^. Vrg4 is a GDP-mannose transmembrane transport in Golgi^38^, and pop6 is a subunit of RNase MRP complex which cleaves pre-rRNA3 and telomerase^39^. The GTPase Bms1 and the mpp10 complex are positioned in the core of the SSU processome. Mpp10 is a component of the small subunit (SSU) processome, required along with imp4 for early co-transcriptional events in ribosome biogenesis^40^. The SSU processome is completed by a centrally placed Rcl1-Bms1 heterodimer and an outer shell of ribosome assembly factors^40^. GTPase bms1 and the endonuclease rcl partake in ribosomal small subunit biogenesis and rRNA processing^41^, as well as the DEAD-box RNA helicase rok1^42^.

Overall, ribosomal biogenesis and rRNA associated processes prevail, with 9 of 13 GO-Slim^43^ annotations referring such terms. In this line, candidate hubs trm112, noc4 and rrp7 are involved in ribosome biogenesis and export, and located in the nuclear compartment of the cell^44–46^. These genes displayed expression correlation with a set of 20 genes enriched for the GO_BP ribosome biogenesis (SPELL analysis ACS > 5.3, *p*-value = 7.31e-23)^47^. Finally, six of the εA surfaced hubs (i.e. mpp10, noc4, bms1, rcl1, imp4, and rrp7) have been recently identified as structural key components of the same nucleolar superstructure, the S. cerevisiae SSU processome^48^.

Altogether, the candidate hubs surfaced by εA are convincingly supported as actual hubs on yeast biology. Therefore, the fact that a widely used measure like εM could underscores their interactions indicate there is still room for improvements on how we detect and quantify genetic interactions, specially in the framework of high-throughput data. Noteworthy, that most of the surfaced hubs are involved in ribosomal biogenesis further suggests that not only the overall number of scored genetic interactions may differ when using one or the other interaction measure; rather, that the study of a particular biological processes through the lens of genetic interactions might be significantly biased, just because the scored method used. The magnitude and direction of such bias, their distribution across fitness values, as well as the type of genetic interaction (suppression/masking), are worth of further research.

## Remarks on causality

### On causal interactions

A central tenets of system biology is that properties of complex systems, not predicted from the individual components, can be essential for understanding the function of the system as a whole^3^. The fact that for example, in a given genetic network background, a phenotype strongly depends on the combination of gene variants at two or more loci, suggests that this dependency would be causally and mechanistically implicated, and hence informative of the functional relationship between genes, and the genetic ordering of regulatory pathways. Therefore, a causal analysis require a holistic approach, that situate the interacting factors in its network background. Previous to this stage, the interaction network should be already wired, even in the loose sense permitted by the data. The model we derived provide the inference permitted by the data, of the interactions wiring such networks, without departing from the rules of probability theory.

The kind of interaction data we are focusing does not permit to ask whether two factors interact mechanistically, in the sense of a collection of causal mechanisms, that require component causes to operates^49^. The high throughput screens that produce these data, deliver information of loose interactions networks, that constitute source of simple hypotheses that should be tested by further systematic analysis of mechanistic and functional relevance, complemented with additional knowledge annotated in databases and in the literature.

Because of the very reasons just posed, it is worth to explicit out that what we have been calling “interaction” all around, is not necessarily a causal interaction. Further, we will see analytically why, by testing the interaction of two fully correlated factors, and of substituting one of the factors by a fully correlated partner (see in Correlation and causality and Association and Causality). Notwithstanding, an argument in favor of our model in that respect, is the cancellation of spurious correlations coming from the population structure (see in Propagated susceptibility). Besides being relevant, this cancellation was not previously demonstrated in the derivation of other interactions models, as far as we know.

### Mechanistic interactions

Indices have been proposed for mechanistic interaction tests, for two binary factors and dichotomous effect, under some moderate assumptions. The peril ratio index of synergy based on multiplicativity’ (PRISM), recently proposed^50^, has the same form of the multiplicative in the complement of the effect model (11). This index invokes the no redundancy assumption^51^, asserting that for every subject in the population, there can be at most one arrival event of the unknown components in a sufficiently short time interval. The correspondence of this index with the sufficient-component cause model (causal-pie model) has been demonstrated by Lee^15^, under the assumptions that the exposure status is time-invariant, the follow-up is fully complete, and there is no confounding, selection bias, or measurement error in the study. Rothman’s model^52^ of sufficient and component causes, often described by pie-charts, is one of the most discussed causal models in epidemiology, aiming the elucidation of the possible mechanisms through which multiple exposures interact in causing an outcome. Lee^15^ show that in the complementary log regression, the coefficient of the cross-product term can be used to test for sufficient-cause mechanistic interactions, the same $\delta$ in (11). According to Lee^15^, the model multiplicative in the complement of the effect can also be used to mechanistic interaction inference, provided suitable causal assumptions are realized.

### Propagated susceptibility

A factor $A$ can’t be associated to a disease $E_{B}$ if $\Pr \left( {E_{B} {|}a} \right) = \Pr \left( {E_{B} } \right)$ for $a \in A$. However, a factor without causal connection to an effect can appear spuriously associated to the effect if it is correlated to a causal factor. Suppose $\Pr \left( {E_{B} {|}b} \right)$ is the risk of a mutation $b$ of a gene $B$ casually associated to cancer $E_{B}$. Let $A$ be a gene not causally associated to that cancer, such that $\Pr \left( {E_{B} {|}a b} \right) = \Pr \left( {E_{B} {|}b} \right)$ for every allele $a \in A$. If some selective phenomenon unrelated to the disease introduces structure in the prior distribution of genes, such that $\Pr \left( {a b} \right) \ne \Pr \left( a \right)\Pr \left( b \right)$, we have

22 $$\Pr \left( {E_{B} {|}a} \right) = \mathop \sum \limits_{b \in B} \Pr \left( {E_{B} {|}b} \right)\Pr \left( {b{|}a} \right),{ }\forall a \in A$$

which imply that $\Pr \left( {E_{B} {|}a} \right) \ne \Pr \left( {E_{B} } \right)$, “associating” gene $A$ to the effect $E_{B}$, even when the molecular machinery involved in the disease is not perturbed by this gene. The right side of (22) can be interpreted as the expected risk accounted from the variants of causal factor $B$ in the proportions they co-occur with the innocuous variant $a$ of factor $A$.

Such spurious association arises for example when a “non-causal” locus is in the close proximity (linkage disequilibrium^53^) to a locus causally connected to a given disease, mimicking the frequencies and correlations of the nearby causal locus and their phenotypes.

The prior structure $\Pr \left( {a b} \right)$ of the population spuriously “propagates” the susceptibility of factor $B$ to factor $A$, explaining why genes are often erroneously associated to diseases. According to (12), the terms $\Pr \left( {\overline{E}{|}a} \right)$ and $\Pr \left( {\overline{E}{|}b} \right)$ introduce spurious susceptibilities $\Pr \left( {\overline{E}_{B} {|}a} \right)$ and $\Pr \left( {\overline{E}_{A} {|}b} \right)$ in the numerator of (15). These fake associations cancel with the denominator, according to (13), and the neutral model ends up depending only on the true susceptibility carriers $\Pr \left( {\overline{{E_{A} }} {|}a} \right)$ and $\Pr \left( {\overline{{E_{B} }} {|}b} \right)$, see Eqs. (12)–(14).

### Correlation and causality

Suppose that factor $A$ is fully correlated with a factor $C$ in the sense that for each instance $a \in A$, there is a single instance $c \in C$ such that

23 $$\Pr \left( {a{|}c} \right) = \Pr \left( {c{|}a} \right) = 1, \Pr \left( {a c^{*} } \right) = \Pr \left( {a^{*} c} \right) = 0, a^{*} \ne a, c^{*} \ne c$$

Hence, exposition to $a \in A$ implies exposition to the predetermined partner $c \in C$, and vice versa. All the Eqs. (3)–(10) satisfied in terms of $a \in A$ are equally satisfied by replacing $a$ by the partner $c \in C$. In particular, $\Pr \left( {E{|}a} \right) = \Pr \left( {E{|}c} \right)$ and $\Pr \left( {E{|}a b} \right) = \Pr \left( {E{|}c b} \right)$, hence, $A$ and $C$ are associated to $E$ with the same strength. Notice that these relations involving $E$ happens to be always the case, even when the effect $E$ is not involved in (23). Indeed, these equalities arise whether $A$ or $C$ share or not the same mechanisms. For example, factor $C$ might be causally involved in the activation of some mechanism in a molecular pathway toward the effect, but $A$ is not involved in any pathways perturbing $E$. However, when $a$ is present, the actual activator $c$ is present because of (23), and the pathway is activated not because the former, but because the later. Hence, the actual “causal” factor associated to an effect cannot be asserted or recognized by frequencies observation alone. In every case, another factor like (23), fully correlated to the factor we are observing can be, unknowingly, the actual cause.

Further, whenever (23) is satisfied, exposition to factors $A$ is by all regards, logically equivalent to exposition to $C$, even when they could be physically or biologically different. Not only $\Pr \left( {E{|}a b} \right) = \Pr \left( {E{|}c b} \right)$, but also $\Pr \left( {b{|}c} \right) = \Pr \left( {b{|}a} \right)$ for any other factor $B$, since

$$\Pr \left( {b{|}c} \right) = \mathop \sum \limits_{{a^{\prime}}} \Pr \left( {b{|}a^{\prime}c} \right)\Pr \left( {a^{\prime}{|}c} \right) = \Pr \left( {b{|}a c} \right) = \Pr \left( {b{|}a} \right)$$

Therefore, all the Eqs. (1)–(13), satisfied in terms of $a \in A$, are equally satisfied by replacing $a$ by the partner $c \in C$.

We finish this epigraph by assessing the neutral model with two fully correlated factors. Regarding our requirements for interactions, a partner-pair $a c$, of factors $A$ and $C$ jointly distributed as in (23) satisfy

$$\Pr \left( {\overline{E}{|}c} \right) = \mathop \sum \limits_{x \in A} \Pr \left( {\overline{E}{|}x c} \right)\Pr \left( {x{|}c} \right) = \mathop \sum \limits_{x \in A,y \in C} \Pr \left( {\overline{E}{|}x y} \right)\Pr \left( {x{|}c} \right)\Pr \left( {y{|}a} \right) = \Pr \left( {\overline{E}{|}a c} \right) = \Pr \left( {\overline{E}{|}a} \right)$$

and then

$$\Pr \left( {\overline{E}{|}a c} \right) = \frac{{\Pr \left( {\overline{E}{|}a} \right)\Pr \left( {\overline{E}{|}c} \right)}}{{\mathop \sum \nolimits_{x \in A,y \in C} \Pr \left( {\overline{E}{|}x y} \right)\Pr \left( {x{|}c} \right)\Pr \left( {y{|}a} \right)}} = {\mathcal{N}}\left( {\overline{E}{|}a c} \right)$$

Hence, the necessary condition for no interaction $\Pr \left( {E{|}a c} \right) = {\mathcal{N}}\left( {E{|}a c} \right)$ is satisfied, which does not permit accepting neither rejecting the hypothesis of interaction. So, the full correlation case doesn’t provide evidence for interaction, even when the built-in cleaner removed the propagated susceptibility due to full correlation between factors $A$ and $C$ defined by (23). Allowance to partner diversity is required to be able to detect interaction. In other words, it is required to compare the join effect of one instance of a factor with different instances of the other factor. For example, to gather information on inhibition or enhancement, at least the effect of the pairs $a c$ and $a c^{*}$ are required. Thus, interventions that breakup this prior correlation are required, otherwise no data size will provide information for interaction between fully correlated factors.

### Association and causality

**Figure 4:**
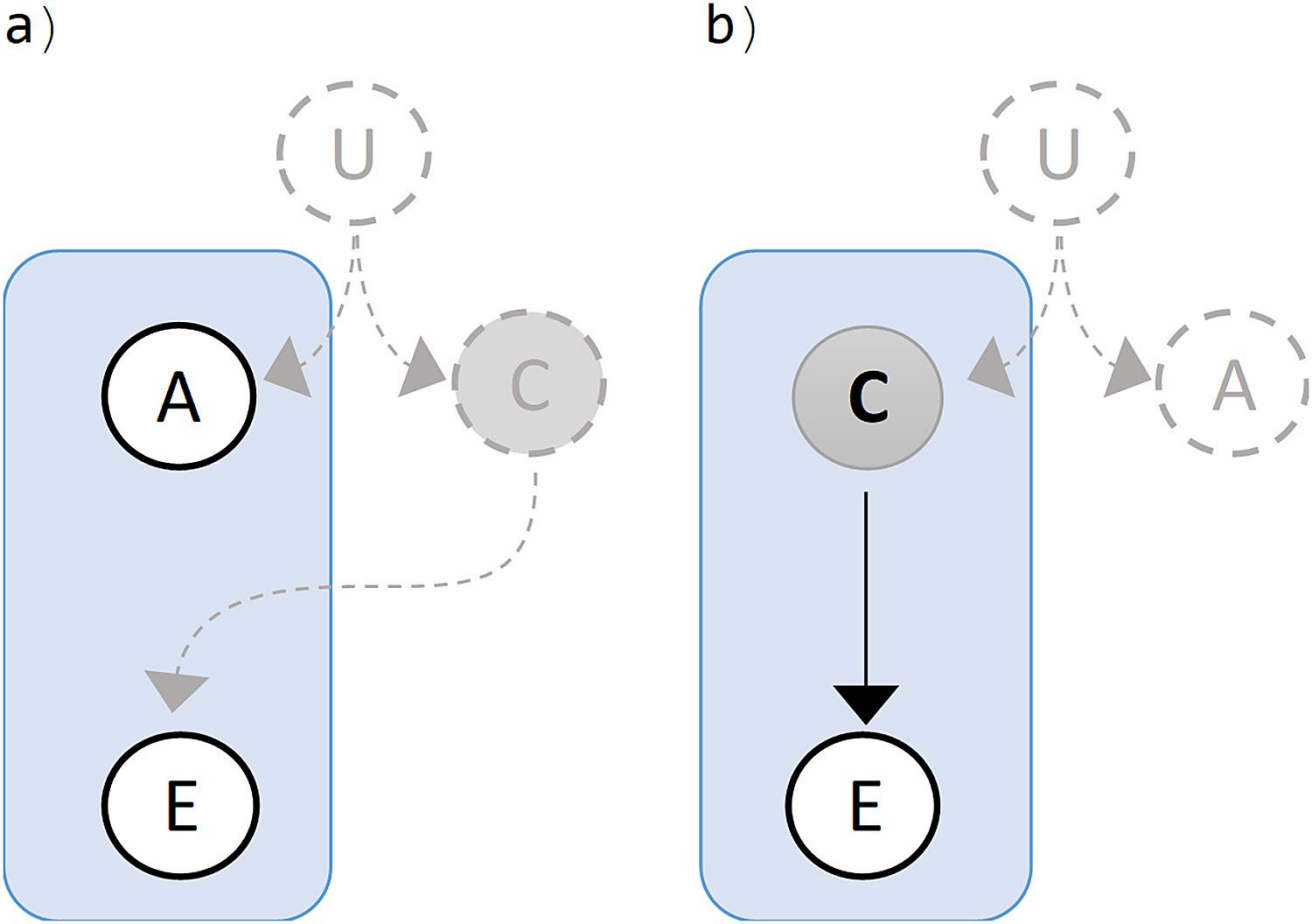
Assessing association of non-causative factor A and causative factor C with the effect E. Unknown mechanism U force the correlation between A and C. (**a**) Spurious association of factors A to the effect E, where factors C is not observed. (**b**) Causative association of factors C to the effect E, where factors A is not observed. (Draw with PowerPoint^23^).

The full-correlation regime (23) is an extreme circumstance used to magnify the issue, but it is a realizable one. Consider for example the case of two genes $A$ and $C$ in a recently in-breeding population with alleles variant $a,a^{\prime}$ and $c,c^{\prime}$ respectively. Suppose that gene $C$ is an oncogene with a malign mutant variant $c^{\prime}$ involved in a cancer disease $E$, gene $A$ is involved in other pathways unrelated to this disease, and $A$ and $C$ are not sharing any regulation mechanisms. Suppose genes $A$ and $C$ are strongly linked in the same chromosome, such that from the four possible haplotypes, only $a c$ and $a^{\prime} c^{\prime}$ are circulating in the population. Gene $A$ can be an optimal marker for the disease, but for example, inducing mutation in variant $a$, or knocking down/out the gene, will only cause perturbations not related to the disease. On the other hand, interventions in the gene $C$ will modify the oncogenic effect of the malign variant allele $c^{\prime}$. No causal model can differentiate gene $A$ from $C$, from the incident of factors and effect alone, by the very reason that $A$ mimics every counting statistics of $C$, and we don’t have further assumptions and information at this stage. The association scenario is sketched in Fig. 4a,b, where U enforced the correlation between genes A and C, and the sole information available to us at this stage is enclosed in the rectangles.

**Figure 5:**
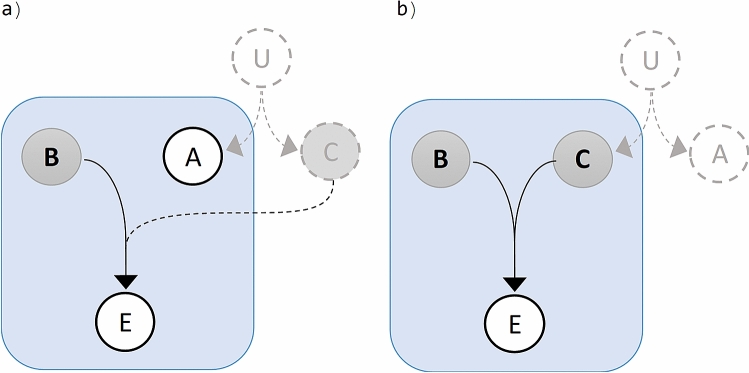
Interaction data of binary factors dichotomous effect E. Unknown mechanism U force the correlation between A and C. Factors B and C are jointly causative, while A is not causative. (**a**) Assessing the interaction of factors A and B, where factors C is not observed. (**b**) Assessing the interaction of factors A and C, where factors A is not observed. (Draw with PowerPoint^23^).

Now suppose the causal gene $C$ require a defect in a tumor suppressor gene $B$, so as to jointly produce a definite oncogenic effect. Some mechanism $U$ hidden to us (Fig. 5), constricts the exposition to the variants of factors $A$ and $C$ according to the correlation in (23), but only $C$ is a causative factor. The scenario is depicted in Fig. 5, where the sole information available to us is enclosed in the rectangles. The resolution of the hypothesis of interaction between factors $C$ and $B$, (Fig. 5a), can not be distinguished from the resolution of the hypothesis of interaction between factors $A$ and $B$, (Fig. 5b). No model for interaction can differentiate gene $A$ from $C$ regarding their interaction with $B$, from the incident of factors and effect alone, by the very reason that $A$ mimics every counting statistics of $C$, and we don’t have further information at this stage.

Simply, when a causal or mechanistic hypothesis is not there, and if we don’t put it in, no amount of sample data increase will provide it^54^. However, large-scale experiments nowadays are examples of mass-discoveries without a priori possession of such causal hypothesis at this preliminary stage of network wiring. It works because the association-to-A hypothesis (Fig. 5a) can take us closer to the causative factor C, since we can go further for knowledge related to A to rise alternatives hypotheses. We can find some annotated experiment where A has been activated as consequence of some perturbation or mechanism U, and find in another report some association of C to U, and so on, so as to gather alternative hypothesis, the actual one among them. However, all these researches are posterior to the initial wiring stage, where our information is limited to what is enclosed in the rectangles, but with the potential to drive our future attention through association, to relevant few hypotheses among the combinatorial multitude of irrelevant possibilities.

Further experimental designs of interventions and/or alternative sources of information are required to inquire about the causal natures of phenomena. Synthetic genetic arrays^1^, and the various technologies of genome wide editing, can provide such interventions for gathering relevant information, and, delivering and testing causal hypotheses. These technologies have revolutionized biomedical research with promising impact in the clinic. However, their extensive use has revealed much unpredictability. The variability in genome editing outcomes remain a challenge, and both on-target efficiency and off target effects are the major concern^55,56^. These large-scale editing tools can produce unpredicted correlation, that can resemble scenarios like the one in (Fig. 5). For example, in RNA-guided DNA targeting platform (CRISPR-Cas), sequence similarities with less than perfect identity with the target (A), is the potential for off-target (C) DNA binding, that could permanently disrupt normal gene function and lead to unpredictable effects, incorrectly attributed to the target (A). But again, knowingly, the very nature of the off-target effect, give some cues (sequence similarities) on where to look, when a tentative target (A) turned out strongly associated.

But there is another side of the story, inherent to modelling, that should be differentiated from the correlated situation of Figs. 4 and 5. A model-biased association can be so misleading, that no mathematics, sample size, or previous annotated knowledge, will be able to overcome. A mistaken association due to model bias, does not lead nor connect to any biological o physical meaning, and any further attempt to deliver meaningful hypotheses from the “finding”, will be a waste.

## Conclusions

A measure of interaction was derived solely from a practical definition asserting that two factors interact when the events they trigger cross or interconnect somewhere in the pathways toward the inquired effect. This measure for multivalued factors and dichotomous effect leads to Finney’s independent action principle as a necessary condition for no interaction. This model obtained from basic principles, though accepted in toxicology, is ignored or undervalued by the dominant epidemiological and genetic networks literature. However, the application in epidemiology is straightforward in term of risk, while it needs to be casted into the parameter space of the application domain in other fields. We showed how to cast the model multiplicative in the complement of the effect into the domain of current genome-wide technologies for genetic interaction. The additive measure in growth rates derived from the model has been assessed with real data from large scale fitness experiments on yeast, and the results were supported by biological evidence validated in the literature and from systematically annotated databases. The theoretical properties revealed for the first time in our derivations, asseverate the advantages of this measure not proved in other alternatives. Further, the unity of arguments elaborated here illustrates how dissimilar interaction contexts can be accommodated into a common framework. We hope this unified view contributes towards relaxing the vivid controversy and coexistence of different models of interaction that plague the literature.

## Supplementary information

Supplementary Information 1.

Supplementary Information 2.

## Data Availability

We downloaded the normalized interaction data files SGA_ExE from http://thecellmap.org/costanzo2016/^1^. The normalization removed systematic biases in colony size arising from experimental factors, and a model of fitness and genetic interactions for each double mutant were fit to the normalized colony sizes^1^. For our purpose, entries with NaN in any of the numerical fields or with negative fitness values were ignored. The columns named “Query single mutant fitness (SMF)”, “Array SMF”, and “Double mutant fitness” are here denoted $\lambda_{01}$, $\lambda_{10}$ and $\lambda_{11}$ respectively. The entries were filtered for the analysis by the criterium *p*-values < 0.05 (column *p*-value). No datasets were generated during the current study.
