# Supplementary material for: A self-consistent probabilistic formulation for inference of interactions
Source: Sci Rep. 2020 Dec 8;10:21435. doi: 10.1038/s41598-020-78496-8 (PMC7722874; doi:10.1038/s41598-020-78496-8)
Supplement: Supplementary file 1 — Supplementary Information 1. [file 41598_2020_78496_MOESM1_ESM.pdf]

A SELF-CONSISTENT PROBABILISTIC FORMULATION FOR INFERENCE OF INTERACTIONS

Jorge Fernandez-de-Cossio[*1], Jorge Fernandez-de-Cossio-Diaz[2], Yasser Perera[3]

[1]Bioinformatics Department, Center for Genetic Engineering and Biotechnology (CIGB), PO Box 6162, CP10600, Havana (Cuba).

[2]Systems Biology Department, Center of Molecular Immunology, PO Box 6162, CP10600, Havana (Cuba).

[3]Molecular Oncology Group, Pharmaceutical Division, Center for Genetic Engineering and Biotechnology (CIGB), PO Box 6162, CP10600, Havana (Cuba).

*To whom correspondence should be addressed jorge.cossio@cigb.edu.cu.

Supplementary Information

An equality derived from the requirements

An analog relation to requirements (i) of requirements (1) can be derived for E_B from the standard probability rules and using (ii),

$$\Pr(E_B|a\ b) = \Pr(E_B|E_A\ a\ b)\Pr(E_A|a\ b) + \Pr(E_B|\overline{E_A}\ a\ b)\Pr(\overline{E_A}|a\ b)$$

and substituting $\Pr(E_B|E_A\ a\ b) = \Pr(E_B|b)$ and $\Pr(E_B|\overline{E_A}\ a\ b) = \Pr(E_B|b)$ from (ii)

$$\Pr(E_B|a\ b) = \Pr(E_B|b)\{\Pr(E_A|a\ b) + \Pr(\overline{E_A}|a\ b)\}$$

yielding

$$\Pr(E_B|E_A\ a\ b) = \Pr(E_B|b)$$

Subtract from one (apply 1 minus to) both side of the above equality, yields the same relation for $\overline{E_B}$:

$$\Pr(\overline{E_B}|E_A\ a\ b) = \Pr(\overline{E_B}|b) \quad (1)$$

Multiplicative on $\boldsymbol{E}$ vs. on $\overline{\boldsymbol{E}}$

The "interaction" terms in the functions $p_{xy} = \exp(\mu + \alpha x + \beta y + \delta xy)$ and $1 - p_{xy} = \exp(\mu' + \alpha' x + \beta' y + \delta' xy)$ for $x, y \in \{0,1\}$, can be obtained by

$$\delta = \log\frac{p_{01}\,p_{10}}{p_{00}\,p_{11}}, \qquad \delta' = \log\frac{(1-p_{01})(1-p_{10})}{(1-p_{00})(1-p_{11})} \quad (2)$$

provided $p_{xy} > 0$. To visualize the magnitude of the departure, we generated 100 000 sets of four random numbers in the range $(0,1)$, corresponding to p_{00}, p_{01}, p_{10} and p_{11}. The interaction terms were computed according to (2). The results are shown in the plot of Figure S 1. It is evident that, in general, δ and δ' differs, and can even be different in sign.

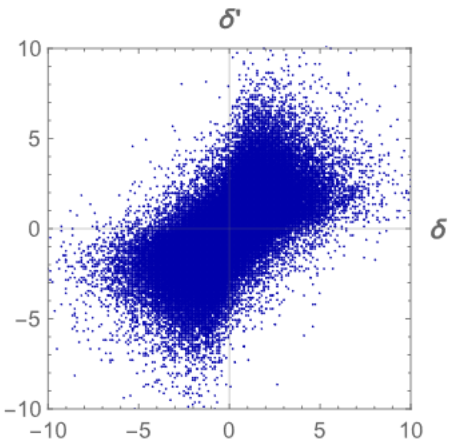

Figure S 1: Distribution in the plane of the interaction terms $\{\delta, \delta'\}$ *computed according to (2), obtained from* $100\,000$ *sets of four random numbers in the range* $(0, 1)$*, corresponding to* p_{00}*,* p_{01}*,* p_{10} *and* p_{11}*.*

We bring here a case example where the prescription of both, δ_{xy} and δ'_{xy}, coincide in an obvious interaction scenario. Suppose that healing E can be realized only in the join combination $E_A E_B$ of the effects of drugs a and b, respectively. It means no healing is achieve from the administration of one of the drugs alone, and discovering that both in combination heals became surprising. An analog case, synthetic lethal are variants at different loci that have little or no effect on viability singly but cause lethality in combination. In the first case E is heal, in the other E is death. They are interaction cases since we need the join combination of the factors a and b for the realization of effects $E_A E_B$, which is the sole combination that realize E. From the description of the problem, the probability of the other five possibilities in the left column of Table 1 has similar probabilities and are vanishingly small. Then

$$\Pr(E|a\,b) \approx \Pr(E_A E_B E_Z|a\,b) + \Pr\left(E_A E_B \overline{E_Z}|a\,b\right) = \Pr(E_A E_B|a\,b)$$

By the statement of this interaction scenario, the combined effects $E = E_A E_B$ are favored by exposure to ab and not by the others exposures $a\overline{b}$, $\overline{a}b$ and $\overline{a}\overline{b}$, so it is expected that

$$0 < p \approx q \approx r \ll \Pr(E|a\,b) \tag{3}$$

where we denoted $p = \Pr\left(E\middle|a\,\overline{b}\right)$, $q = \Pr(E|\overline{a}\,b)$ and $r = \Pr\left(E\middle|\overline{a}\,\overline{b}\right)$. From the statement of the problem, both factors a and b are required to realize E, hence, the probabilities p, q, and r are small but very similar, so, $pq/r \approx p$. The model multiplicative in the effect assert that on absent of interaction $pq/r = \Pr(E\,\middle|\,a\,b)$. But $pq/r \approx p \ll \Pr(E\,\middle|\,a\,b)$, indicating interaction.

On the oder side, the model multiplicative in the complement of the effect assert that on absent of interaction

$$\frac{(1-p)(1-q)}{1-r} = 1 - \Pr(E|a\,b)$$

But $(1-p)(1-q)/(1-r) \approx 1-p$ from the stamen of the problem, then $(1-p)(1-q)/(1-r) \gg 1 - \Pr(E|a\,b)$, indicating interaction.

More on gene-hubs

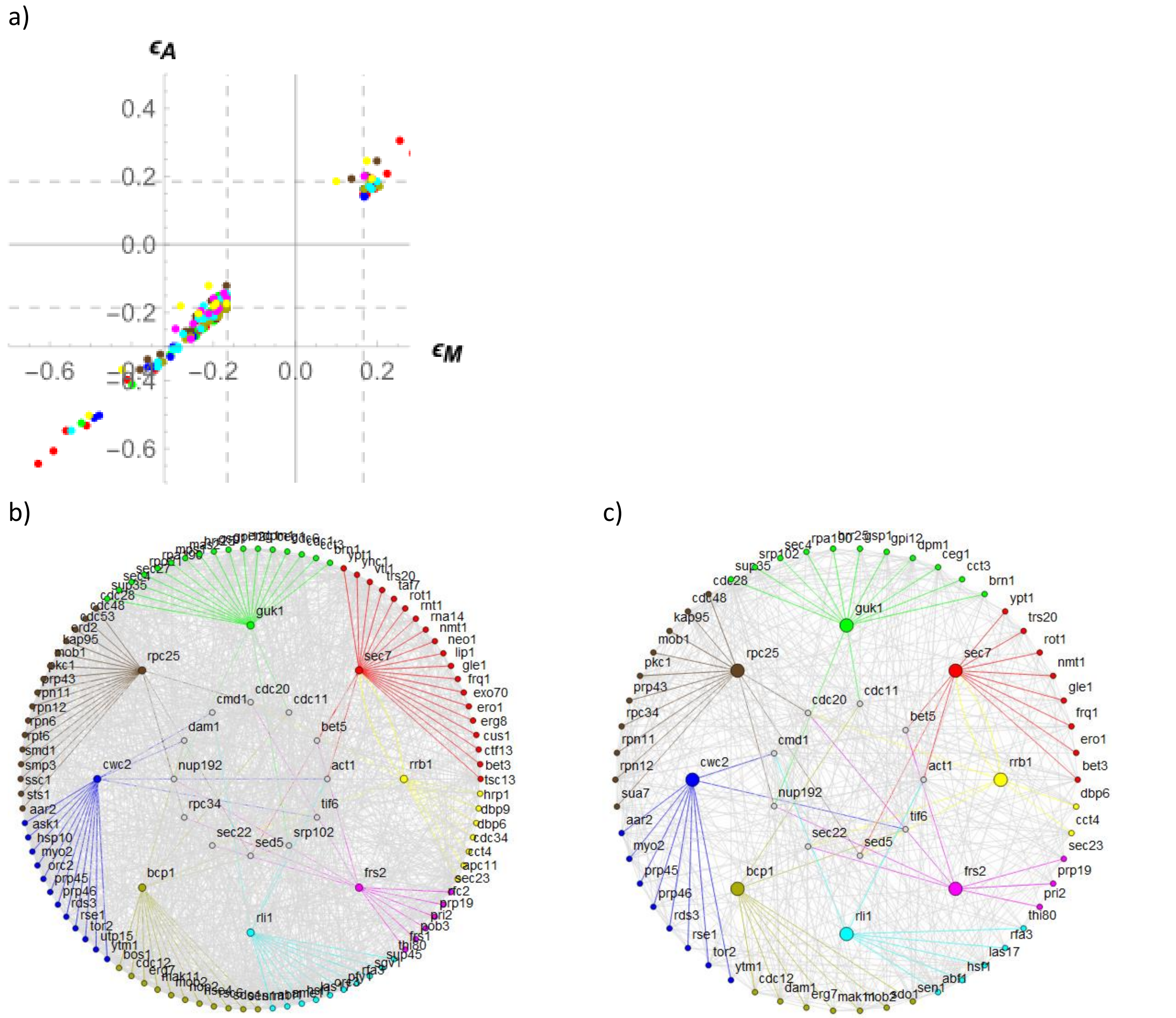

Figure S 2: a) Comparison of the interactions scores ϵM and ϵA for candidate hubs of Table S 1. b) c) Interaction network, including the connections between the interactors. The hubs are located in the middle ring with larger dots. The interactors that are not connected to more than one hub are in the outer ring. The rest of interactors are in the inner ring. The hub-connections has the same color of the corresponding hubs. The other connections are in light gray. b) Interaction network as computed by ϵM. c) Interaction network as computed by ϵA. The dot colors are consistently used.

Table S 1: List of genes predicted by ϵM with more than 1.5 folds the interactors predicted by ϵA (interaction criteria ϵ > 2 s.d.).

candidate hubs	# of interactors (ϵM)	# of interactors (ϵA)	# of common interactors
sec7	23	12	12
guk1	20	13	13
rpc25	20	13	12
cwc2	16	10	10
bcp1	15	9	9
rli1	13	7	7
frs2	11	7	7
rrb1	10	6	5
